# Highbush Blueberry (*Vaccinium corymbosum* L.) Leaves Extract and Its Modified Arginine Preparation for the Management of Metabolic Syndrome—Chemical Analysis and Bioactivity in Rat Model

**DOI:** 10.3390/nu13082870

**Published:** 2021-08-20

**Authors:** Oleh Koshovyi, Sebastian Granica, Jakub P. Piwowarski, Oleksandr Stremoukhov, Yuliia Kostenko, Ganna Kravchenko, Oksana Krasilnikova, Andriy Zagayko

**Affiliations:** 1 Department of Pharmacognosy, National University of Pharmacy, 53 Pushkinska Str., 61002 Kharkiv, Ukraine; oleh.koshovyi@gmail.com (O.K.); astrapharm1971@gmail.com (O.S.); kostenkou567@gmail.com (Y.K.); 2 MicrobiotaLab, Department of Pharmacognosy and Molecular Basis of Phytotherapy, Faculty of Pharmacy, Medical University of Warsaw, Banacha 1, 02-097 Warsaw, Poland; jpiwowarski@wum.edu.pl; 3 Department of Biological Chemistry, National University of Pharmacy, 53 Pushkinska Str., 61002 Kharkiv, Ukraine; annabk2014@gmail.com (G.K.); krasilnikovaoksana16@gmail.com (O.K.); andrey.zagayko@gmail.com (A.Z.)

**Keywords:** *Vaccinium corymbosum*, leaves, extract, phenolics, arginine, metabolic syndrome

## Abstract

Growing blueberry (*Vaccinium corymbosum* L., Highbush blueberry) as a berry crop is developing dynamically, especially in warm temperate, subtropical, and tropical regions of the world. When blueberry is cultivated on plantations, the bushes are pruned annually, and tons of leaves become waste. Thus, the aim of the present study was to create a preparation from blueberry leaves, study their chemical composition and determine their potential as a dietary supplement for the prophylactic and correction of the metabolic syndrome. Several schemes for obtaining extracts from blueberry leaves have been developed, including one with addition of arginine. A total of 18 phenolic substances were identified and quantified in the extracts by TLC and HPLC methods. Chlorogenic acid, hyperoside, and rutin were shown to be dominating constituents. Quantitative determination of hydroxycinnamic acid derivatives, flavonoids and other phenolics in the extracts was performed by spectrophotometric method. The extracts administration led to a significant decrease in the level of glucose, insulin and triacylglycerols in blood serum of adult mature inbred rats with insulin resistance induced by the fructose-enriched diet. The most promising one was the extract modified with arginine. The determined hypoglycemic and hypolipidemic activity of chemically standardized extracts from highbush blueberry leaves indicate the potential of this crop residue in utilization as a dietary supplement recommended in prevention of ailments associated with metabolic syndrome.

## 1. Introduction

Metabolic syndrome (MS) is a cluster of abnormalities combining insulin resistance (IR), obesity, hypertension, atherosclerotic hyperlipidemia, and some other metabolic disorders [1]. From 1998, the World Health Organization (WHO, Geneva, Switzerland) first defined MS (or Syndrome X, or insulin resistant syndrome) and published criteria. Several different definitions were proposed but all of them include glucose intolerance, IR, dyslipidemia, and hypertension [2]. Currently, WHO experts suggest MS to be a pandemic [3,4,5,6]. Though there are gender-, age- and race-based variations, one in four citizens in developed countries suffers from MS. Over the next 25 years, an increase in the rate of expected incidence is 50% [7]. Additionally, MS is strongly associated with diabetes mellitus type 2 (DM2) and cardiovascular diseases (CVD), which are the leading cause of mortality [8].

In Ukrainian traditional medicine, shoots and leaves of the bilberry (*Vaccinium myrtillus*), which belongs to of the genus *Vaccinium* of the Heather family (*Ericaceae*), are widely used, as a hypoglycemic agent. In the form of a decoction or raw materials, these are included in herbal teas with sugar-lowering properties such as Arfazetin and Mirfazin [9]. It was also shown that administration of the common blueberry leaves dry extract has a normalizing effect on metabolic disorders under the high-fructose diet. The revealed effects were caused by the hypoglycemic, hypolipidemic, and antioxidant properties of extract components [10,11].

Genus *Vaccinium* contains more than 200 species. We suppose that the plant materials of other species belonging to this genus could represent interesting biological properties which could be utilized in MS prevention. Herbal raw materials of blueberries *Vaccinium uliginosum* L., a wild plant species, and *Vaccinium corymbosum* L., which is most widely cultivated, could be of particular interest [12].

Growing blueberry (*Vaccinium corymbosum* L., highbush blueberry) as a berry crop is developing dynamically, especially in warm, temperate, subtropical and tropical regions of the world [13,14]. As of 2017, the area of planting highbush blueberry was 341 thousand hectares in the world. Commercial cultivation of highbush blueberry growth started only in 2007, however, and as of 2015, the area under this crop already accounts for a total planting area of 700–750 ha in Ukraine. The yield of blueberry fruits is about 7.4–7.6 t/ha. The main regions of commercial cultivation of highbush blueberry in Ukraine are Ivano-Frankivsk, Volyn, Vinnytsia, Zhytomyr and Kiev regions [15].

Blueberry fruits (*Vaccinium corymbosum* fructus) are widely used in traditional medicine, pharmacy and the food industry [16,17,18]. The berry is nutritious and has sweetish taste. Juices, compotes, jams, wines, jams, jellies and mousses are made from berries. Berries are used fresh, frozen or dried [19]. The leaves and dried fruits are used for making herbal tea [20].

There are many dietary supplements containing highbush blueberry preparations on the Ukrainian market. In other countries, preparations from the blueberry fruits are used to improve vision. The dietary supplement “Golubitoks”, a concentrate made from berries and leaves of blueberries, has anti-inflammatory and pronounced antiviral effects, improves the immune system and normalizes its functioning, and is used for supportive treatment of hypertension [9,21]. Blueberry fruit and juice are dietary products that enhance metabolism and action of sugar-lowering drugs [22,23]. The fruits of this plant are widely used especially by the food industry, hence their well-defined chemical constitution (anthocyanins, vitamins (B1, B2, PP, C, A) and minerals (Ca, P, Fe) [17].

Several studies focused on the evaluation of the chemical composition of *V. corymbosum* were performed. Fifteen anthocyanins were characterized and quantified in V. corymbosum berries and juice. The major compounds detected were delphinidin 3-glucoside and delphinidin 3-galactoside and peonidin 3-arabinoside [24,25]. The *Vaccinium corymbosum* leaf extract was found to be rich in polyphenolic compounds: two phenolic acids—chlorogenic and caffeic acids and two quercetin glycosides—rutin and isoquercitrin were identified by HPLC [26,27,28]. Another study analyzed forty-four flavonols in 30 samples of highbush. Highbush fruits contained mainly quercetin 3-galactoside and quercetin 3-rhamnoside. Additionally, several other compounds were identified including simple myricetin, laricitrin, quercetin, isorhamnetin and syringetin glycosides or their acetylated derivatives. The study also proved the presence of chlorogenic acid in the analysed material [29]. Important groups of compounds identified as part of the berry lipophilic compounds are triterpenes (oleanolic acid, ursolic acid, lupeol and lanosterol), sterols (β-sitosterol) and fatty acids [30]. Most available data focused on the chemical analysis of fruits obtained from *V. corymbosum*. Data on natural products present in leaves of this plant material are still limited and not comprehensive.

When blueberry is cultivated on plantations, the bushes are pruned annually, and tons of leaves become waste, while they contain a significant amount of biologically active substances, which properties could be further utilized as sustainably developed products of plant origin. Thus, the leaves of *V. corymbosum* act as by-products and are an interesting material for the development and manufacturing of food supplements with promising preventive and therapeutical properties.

Modification of biologically active molecules by their conjugation with amino acids is a known strategy for modification of their biological properties. For example, the synthetic medicine Valtrex was created by combining acyclovir with the amino acid valine [9], the medicine L-lysine escinate was created using the modification of the complex of chestnut triterpene saponins (β-escin) with L-lysine [9,31]. Therefore, it has become an interesting question, whether the use of such an approach to modify the total plant extract could result in improvement of its biological properties. It was previously shown that the modification of tincture *Leonurus cardiaca* L. with amino acids has led to the creation of new more active substances with anxiolytic activity [32]. The modification of the blueberry leaves extract with arginine allowed to create a substance with pronounced hypoglycemic and hypolipidemic activities [10]. The modification of the extract of bearberry leaves with phenylalanine allowed to create a substance with a pronounced diuretic and anti-inflammatory effect [33]. All of this indicates the viability of the chosen direction.

Arginine plays a crucial role in the pathogenesis of diabetes due to its activity against dysfunction of the vascular endothelium. The key factor regulating the tone of the vascular endothelium is the most important physiological vasodilator-nitrogen monoxide. This mediator is formed from arginine under the action of Ca^2+^ dependent enzyme endothelial NO-synthase (eNOS) [34,35]. That is why we have chosen arginine as a modifying agent, hypothesizing that the combination of arginine with a plant derived extract is a promising direction of modification of its preventive and/or therapeutic properties.

Several previous studies showed that amino acids present in proteins can interact with polyphenols including chlorogenic acid and flavonoid derivatives. The interaction often results in non-covalent binding of NH_2_ groups of amino acids and OH groups of polyphenols. It was also reported that in some cases covalent adducts including Schiff bases can be formed [36,37].

The aim of our research was to establish the chemical composition of *V. corymbosum* leaves extract and its preparation obtained after the heating with arginine as well as their bioactivity in vivo as potential dietary supplement for the management of MS.

## 2. Materials and Methods

### 2.1. Chemicals and General Experiments

Deionized water was produced using Millipore Simplicty UV station (Merck Millipore, Burlington, MA, USA). Acetonitrile, formic acid, ethanol was purchased from VWR (Radnor, PA, USA). Chlorogenic acid, hyperoside, rutin, gallic acid were purchased from Carl Roth (Karlsruhe, Germany). L-arginine, Tween-80 and aluminum chloride were purchased from Sigma-Aldrich (Sant Louis, MI, USA). Querectin was bought from Borschagovsky CPP (Kyiv, Ukraine) and fructose—from LLC Company ”Ukrhimsyre” (Kharkiv, Ukraine). Blood glucose, high-density lipoprotein cholesterol (Ch-HDL) and low-density lipoprotein cholesterol (Ch-LDL) (Felitis-Diagnostics, Ukraine) insulin (DRG, Germany) and triacylglycerols (TAG, Lachema, Czech Republic) were determined in blood serum using standard sets of reagents. Chemical standards used for HPLC analysis were previously isolated and identified in the Department of Pharmacognosy and Molecular Basis of Phytotherapy, Medical University of Warsaw.

### 2.2. Plant Material 

The leaves of *Vaccinium corymbosum* L., which were obtained from plants harvested in September 2018 on private plantings of highbush blueberry in Sadko Garden Center of Kyiv region (GPS 50.459228, 30.800649). The samples of *Vaccinium corymbosum* L. were identified by prof. T.N. Gontova from the Botany Department, the National University of Pharmacy (Kharkiv, Ukraine), using a special botanical catalog [38]. The voucher specimens are stored at the Department of Pharmacognosy, The National University of Pharmacy, Kharkiv, Ukraine (No. 546–548).

### 2.3. Preparation of Extracts

Five hundred g of dried highbush blueberry leaves [39], ground to a particle size of 1–2 mm, were placed in an extractor, and was macerated with 3 L of ethanol: water mixture (1:1, *v/v*) overnight at room temperature. The extraction was repeated three times with new portions of the solvent (1.0 L). The resulting extracts were combined, settled for 24 h, and filtered through a folding filter. Next, half of the obtained extract was evaporated to dryness (E1) to obtain 102.5 g of the extract. The yield was 41%. 

Arginine (52 g—0.3 mol) was added three times at the equimolar amount to the phenolic compounds (according to determined gallic acid equivalents) to the remaining extract of the highbush blueberry [the content of phenolic compounds in terms of gallic acid was 17.01 g (0.1 mols of gallic acid equivalents) in the remaining volume of the liquid extract]. The resulting solution was heated overnight at 50 ± 5 °C and evaporated using a rotary vacuum evaporator to obtain 140.7 g of dry residue (E2). The yield was 56.3%.

### 2.4. Quantification of Major Phytochemicals Using Non-Specific Chemical Methods

Quantitative determination of hydroxycinnamic acid derivatives, flavonoids, and phenolic compounds in the filtrate in terms of dry residue was performed by spectrophotometric method. The optical density was measured on a Specol 1500 spectrophotometer (Switzerland). The content of hydroxybutyric acid derivatives was determined in terms of chlorogenic acid at 327 nm, the total flavonoid content in terms of routine—at a wavelength of 417 nm after the formation of the complex with aluminium chloride, the content of the phenolic compounds in terms of gallic acid. For statistical validity, the experiments were performed at least five times [40,41,42,43,44,45]

### 2.5. HPLC-DAD-MS Analysis and Quantification of Major Compounds

The HPLC-DAD-MS analysis of extracts E1 and E2 were performed using Ultimate 3000 RS system (Dionex, Sunnyvale, CA, USA) coupled with ion trap mass spectrometer Amazon SL (Bruker Daltonik, Bremen, Germany). The separation was carried on Kinetex XB-C_18_ column (150 mm × 2.1 mm × 1.7 μm, Torrance, CA, USA). The column was eluted with 0.1% formic acid in deionized water (A) and 0.1% formic acid in acetonitrile (B). The gradient program was used 0 min—1% B, 60 min—26% B. The flow rate was 0.3 mL/min and column temperature was kept at 25 °C. The eluate was introduced directly to the ESI source of the mass spectrometer. The ESI source parameters were: nebulizer pressure 40 psi; dry gas flow 9 L/min; dry temperature 300 °C; and capillary voltage 4.5 kV. Compounds were analyzed in the negative and positive ion modes. The MS/MS mode was active and the most abundant ion in the recorded spectrum was subjected to the fragmentation. Signals obtained in MS/MS spectrum were used for further fragmentation whenever possible with Smart Frag mode. Using DAD device, the UV-Vis spectrum of detected compounds were monitored from 200 to 450 nm.

The quantification of detected compounds was performed using negative ion mode of the mass spectrometer. Calibration curves were established for chlorogenic acid (5-*O*-caffeoylqunic acid) and quercetin 3-*O*-galactoside (hyperoside). Mixture of standards was prepared at the concentration of 50 μg/mL for both chemicals. The solution was injected to the HPLC system. Using the mass spectrometer, the analysis was monitored for two sets of characteristic signals. For chlorogenic acid *m/z* = 353 and 707 and for hyperoside *m/z* = 463 and 927 corresponding to [M-H]^−^ and [2M-H]^−^, respectively. Calibration curves were plotted area under the peak vs. amount of the compound injected to the column in the rage 25 to 800 ng per injection. The following curves were obtained: chlorogenic acid—*y* = −360.39*x*^2^ + 3133799.32*x*; hyperoside—*y* = −13387.79 + 8737090.12*x*. R2 in both cases was above 0.999.

For the quantification 10 mg of each extract (E1 or E2) was dissolved in 1 mL of methanol:water mixture (1:1, *v/v*) in triplicate. Next samples were filtered through 0.45 μm PVDF filters and were injected to the HPLC system. Detected compounds were classified to one of two phytochemical groups (phenolic acids derivatives or flavonoids) in the case of E1 and suitable standard was used for the quantification. In the case of E2, all compounds were calculated as chlorogenic acid equivalents. Ions used for quantification and proper chemical standard are given in Table 1 and Table 2.

### 2.6. The Anti-MS Potential of Extracts Using In Vivo Rat Model

The study was conducted according to the guidelines of the Declaration of Helsinki, and approved by the Ethics Committee of the National University of Pharmacy (Protocol #3 from 10.09.2020; approval #3/10092020)). The experiment was carried out in accordance with the International Principles of the European Convention for the Protection of Vertebrate Animals Used for Experimental and Other Scientific Purposes.

The hypoglycemic activity of the dry extract from the leaves of highbush blueberry with the addition of arginine was studied in adult mature inbred rats. Male adult Wistar rats with body weight of 200 ± 15 g were used. Animals were fasted for 12 h prior to experiments. Insulin resistance (IR) and dyslipidemia as components of MS was modelled by the treating animals on a fructose-enriched diet (60.3% fructose, 18.3% protein, 5.2% fat) [34,35]. The experimental animals were randomized into groups (*n* = 6): (1) intact animals, which were kept on a standard diet of vivarium NUPH (Intact); (2) animals kept for six weeks on a high-fructose diet (HFD); (3) animals that were kept for six weeks on HFD and which, beginning from the fourth week, were administered emulsion (Tween-80) of highbush blueberry dry extract E1 intragastrically 150 mg/kg body weight (HFD_E1_150), 250 mg/kg body weight (HFD_E1_250) and 350 mg/kg body weight (HFD_E1_350) every day for two weeks; (4) animals that were kept for six weeks on HFD and which, beginning from the fourth week, were administered daily, intragastrically for two weeks an emulsion (Tween-80) of highbush blueberry leaves dry extract E2 (modified with arginine) at a dose 150 mg/kg body weight (HFD_E2_150), 250 mg/kg body weight (HFD_E2_250) and 350 mg/kg body weight (HFD_E2_350) every day for two weeks; (5) which were kept for six weeks on HFD, and they were administered L-arginine a solution of at a dose of 250 mg/kg body weight (HFD_Arg) intragastrically, starting from the fourth week, daily for two weeks; (6) animals that were kept for six weeks on HFD and who, beginning from the fourth week, were administered intragastrically an emulsion (Tween-80) quercetin daily at a dose of 50 mg/kg body weight (HFD_Q).

The animals were decapitated under thiopental anaesthesia at the end of the sixth week. The blood serum was collected and subjected for further study.

### 2.7. Statistical Analysis

The mean and standard deviation (SD) of the sample were calculated according to the monograph *“Statistical Analysis of the Results of a Chemical Experiment”* of the State Pharmacopoeia of Ukraine, 2.2. The average sample μ was calculated as the arithmetic mean of all variants (*n* = 5 of combined samples). At the same time, the spread of options around the average is characterized by the magnitude of the standard deviation s. The uncertainty of this estimate is characterized by the value of the confidence interval, in which the true value μ is given with the given two-way probability P2. Under uncertainty, the confidence interval is understood, usually for the 95% significance level. Limit values of the confidence interval were calculated using Student’s criterion. Quantitative data are presented as the mean ± SD. level of statistical significance was set at not more than *p* < 0.05 [43,45].

## 3. Results

### 3.1. Quantification of Major Phytochemicals Using Chemical Methods

The simple chemical methods were used for the comparison of the content of hydroxycinnamic acids derivatives, flavonoids and total phenolics. The obtained results are shown in Table 3.

The content of quantified phytochemicals varied from 2.92 to 18.42% in E1 and from 1.82 to 12.09% in E2. In all cases the addition of L-arginine to the extract and heating caused decrease in the amount of each group of compounds. Both extracts showed higher content of flavonoids (3.03% and 1.96% for E1 and E2, respectively) than hydroxycinnamic acids (2.92% and 1.82% for E1 and E2, respectively). The total phenolics content was 18.42% for E1 and 12.09% for E2. Both extracts showed lower total phenolics amount than similar preparations obtained previously from fruits of different *Vaccinium* species [46]. However, measured contents are significantly higher than in the case of previously investigated extracts prepared from the skins of fruits from two berry species [47].

### 3.2. Phytochemical Analysis of Investigated Extracts and Quantification of Major Compounds Using HPLC-DAD-MS Approach

**Figure 1:**
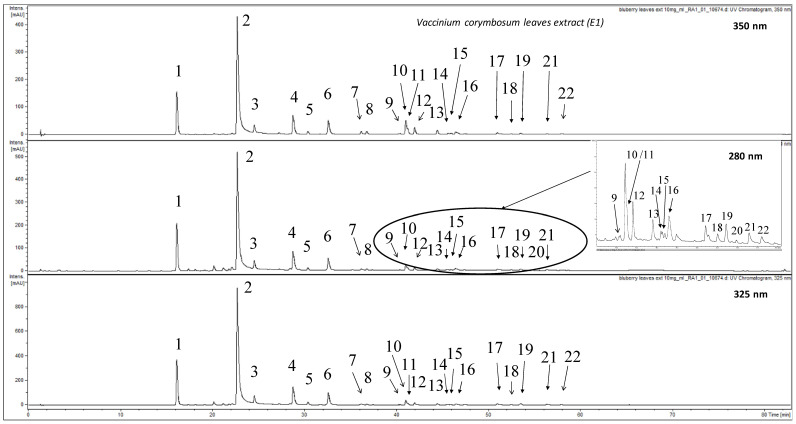
HPLC-DAD-MS chromatogram of E1 recorded at 350, 280 and 325 nm.

The HPLC analysis of the raw extract (E1) prepared from leaves of *Vaccinium corymbosum* led to the detection of 22 compounds (Figure 1, Table 1). Based on recorded data the compounds were classified into respective phytochemical group. In this section the identification was conducted based on MS signals occurring in negative ion mode. Additionally, Table 1 contains data recorded in positive ion mode.

Compounds **1–6**, **17** and **19–21** were preliminary identified as phenolic acid derivatives based on the observed UV-Vis maxima at ca. 240 and 310–325 nm. Compounds **1–3** showed pseudomolecular ion at *m/z* = 353. The fragmentation of this ion led to the base peak in the MS^2^ spectrum at *m/z* = 191 for **1**/**2** and at *m/z* = 173 for **3**. Based on the comparison of fragmentation patterns with available literature and comparison of retention times with chemical standards compounds **1–3** were identified as 3-*O*-, 5-*O*- and 4-*O*-caffeoylquinic acids, respectively [48]. Compounds **4** and **6** showed signals at *m/z* = 439. The fragmentation of this ion led to the production of major peak at *m/z* = 395 corresponding to fast decarboxylation [M-44]^−^. Additionally, the signal from caffeoylquinic acid moiety was observed at *m/z* = 353. After the comparisons of the obtained data with literature both compounds were assigned as isomeric O-malonyl-O-caffeoylquinic acids [49]. However, the full structure elucidation of **4** and **6** was not possible due to the lack of chemical standards. Compound **5** showed based peak ion in the MS spectrum at *m/z* = 335. The further fragmentation led to the production of major fragment at *m/z* = 179, corresponding to caffeic acid residue. The comparison of the recorded data allowed identification of **5** as *O*-caffeoylshikimic acid isomer most probably 3-*O* or 4-*O* derivative [50]. Compound **17** displayed pseudomolecular ion at *m/z* = 515. The fragmentation led to the occurrence of major io at *m/z* = 173. The comparison of the fragmentation profile with literature allowed identification of **17** at 4, 5-*O*-dicaffeoylquinic acid [48]. Compound **21** with base peak ion at *m/z* = 499, showed fragmentation pattern with major ions at *m/z* = 337 and 173. Additionally, the UV-Vis maximum at ca. 315 nm suggested the presence of *p*-coumaroyl moiety in the structure of **21**. The comparison with literature data allowed assignment of **21** as *p*-coumaroyl-caffeoylquinic acid isomer [50]. During the analysis, two other compounds (**19** and **20**) were preliminary identified as phenolic acid derivatives based on their UV-Vis spectra. However, the analysis of their MS spectra did not allow their further assignment.

Compounds **7–16**, **18** and **22** were assigned as flavonoids or their derivatives based UV-Vis maxima observed at ca. 260 and 350 nm. Compounds **7**, **8**, and **18** showed base peak ions in their MS spectra at *m/z* = 479, 479 and 625, respectively. The fragmentation of these ions led to the observation of aglycone moiety at *m/z* = 317 for all three chemicals. They were assigned as myricetin derivatives. The calculation of the neutral loss for 7 and 8 between source ion and the fragment [M-162] suggested that both flavonoids are hexosides. The comparison with chemical standards allowed identification of **7** and **8** as: myricetin 3-*O*-galacotoside and 3-*O*-glucoside. In the fragmentation pattern of 18 a strong signal of a fragment at *m/z* = 479 was also detected as a product of a cleavage of moiety with mass of 146. The additional maximum in the UV-Vis spectrum at ca. 315 nm suggested that the cleaved moiety could be *p*-coumaric acid residue. The obtained data allowed assignment of **18** as mirycetin *p*-coumaroylhexoside.

Compounds **10–13**, **15** and **22** showed in their fragmentation patterns, a strong signal at *m/z* = 301 corresponding to aglycone moiety. Thus, they were assigned as quercetin derivatives. Compounds **10** and **12** had pseudomolecular ions at *m/z* = 463. The further fragmentation showed a cleaved of hexose moiety leading to aglycone signal [M-162] = 301. In the case of **9**, **11** and **22** base peak ions in the MS spectrum were observed at *m/z* = 609. The fragmentation showed a loss of neutral fragment of 146 leading to the occurrence of signals at *m/z* = 463 for all three compounds. Further fragmentation showed cleavage of hexose moiety (−162) and the production of aglycone residue at *m/z* = 301. The comparison with chemical standards allowed identification of **10**, **11** and **12** as quercetin 3-*O*-galactoside, quercetin 3-*O*-rutinoside and quercetin 3-O-glucoside, respectively. The fragmentation of 9 and 22 suggested both compounds could be quercetin *O*-rhamnohexosides. However, in the case of **22** an additional maximum in the UV-Vis spectrum at ca. 315 nm and the high value of the retention time (ca. 58 min) indicated that the residue with the mass of 146 is not a rhamnose but *p*-coumaroyl moiety. Thus **22** was assigned as quercetin *p*-coumaroylhexoside whereas **9** was identified as isomeric to rutin quercetin *O*-rhamnohexoside [51]. Compound **13** showed pseudomolecular ion at *m/z* = 433. The fragmentation revealed the presence of pentose moiety linked to the aglycone (neutral loss of 132 was observed). The comparison with the chemical standard proved that **13** is quercetin 3-*O*-arabinoside (avicularin). Compound **15** had base peak ion at *m/z* = 549. The fragmentation led to the presence of strong signal at *m/z* = 505 after the fast decarboxylation (−44) of the parental ion and the occurrence of aglycone signal at *m/z* = 301. The selective decarboxylation is characteristic for the presence of maloyl unit in the chemical structure of **15** similar to previously described compounds **4** and **6**. The observed MS data followed by calculation led to the conclusion that **15** is quercetin *O*-malonylhexoside.

The analysis showed that the extract also contained two kaempferol derivatives (**14** and **16**). Both compounds showed characteristic aglycone ion signal at *m/z* = 285 in their fragmentation spectra. Compound **16** had base peak ion at *m/z* = 447 which during the fragmentation cleaved hexose unit (−162). The comparison with the chemical standard allowed identification of **16** as kaempferol 3-*O*-glucoside. Compound **14** displayed major ion in the MS spectrum at *m/z* = 593. The fragmentation showed signals at *m/z* = 447 (M-146) and *m/z* = 285 (M-146-162). Due to the lack of additional maximum in the UV-Vis spectrum typical for *p*-coumaric acid derivatives compound **14** was assigned as kaempferol *O*-rhamnohexoside.

The quantification of compounds occurring in the raw extract was performed using MS detection. The ions used for the calculation of the content of each chemical are listed in Table 1. The results show that the extract contains significant amounts of caffeoylquinic acid derivatives. The dominating compound was chlorogenic acid (**2**, ca. 136.5 μg/mg) followed by neochlorogenic acid (**1**, ca. 29.5 μg/mg). It should be noted that the extract also contained significant amounts of malonyl-caffeoylquinic acids (**4** and **6**) which were detected as ca. 17.9 and ca. 13.1 μg/mg, respectively. Other phenolic acid derivatives were quantified as minor compounds (Table 1). The second group of phytochemicals occurring in the analyzed extracts were flavonoids. Their content was significantly lower compared to caffeoylquinic acids. The major flavonoid occurring in the extract was hyperoside (**10**, ca. 4.3 μg/mg). Apart from that high content of isoquercitrin (**12**, ca. 2.3 μg/mg) and avicularin (**13**, ca. 1.2 μg/mg) was established.

**Figure 2:**
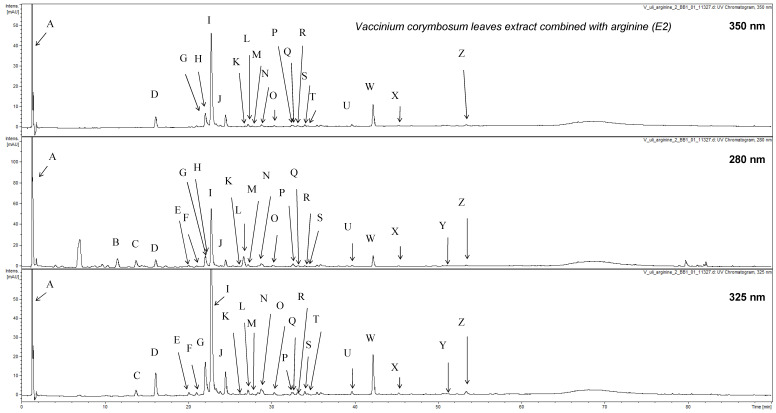
HPLC-DAD-MS chromatogram of E2 recorded at 350, 280 and 325 nm.

The HPLC analysis of the extract heated with arginine (E2) led to the detection of 25 compounds (Figure 2, Table 2).

Compounds were assigned based on their UV-Vis spectra and MS profiles. The analysis showed that heating with the addition of arginine in ethanol led to the significant alteration of the chemical composition of the extract. It could be observed that extract (E2) contained only several native compounds namely chlorogenic acids (**D**, **I** and **J**). Some decomposition products could be detected like quinic acid (**A**) with pseudomolecular ion at *m/z* = 191 and caffeic acid (**G**) showing dominating base peak ion in the MS spectrum at *m/z* = 179. The reaction with ethanol used as a solvent led to the production of four isomeric ethyl esters of caffeoylquinic acids (**S**, **U**, **W** and **X**) with pseudomolecular ions at *m/z* = 381 and [M-OC_2_H_5_]^−^ fragments occurring in fragmentation spectra at *m/z* = 335. The presence of ethyl esters of dicaffeoylquinic acid was also confirmed as compounds **Q** and **R** showing base peak ions in their MS spectra at *m/z* = 543. The cleavage of ethyl moiety and one caffeic acid residue from **Q** and **R** led to the observation of caffeoylquinic acid moiety signal at *m/z* = 353. The chemical alteration also resulted in the occurrence of two additional caffeoyl shikimic acid isomer (**P** and **T**) apart from the compound detected in the native extract (**O**). Compound **E** which was not seen in the native extract was identified as 3-*O*-*p*-coumaroylquinic acid based on the comparison of the obtained MS spectra with suitable literature [48]. Three compounds (**H**, **K** and **L**) were identified as possible conjugates of caffeoylquinic acids and arginine added to the raw extract. All three compounds showed pseudomolecular ions at *m/z* = 527. The fragmentation of these ions led to the observation of caffeoylquinic fragments at *m/z* = 353. The calculation of the neutral loss suggested the presence of arginine residue in the chemical structure of **H**, **K** and **L**. Apart from compounds that were preliminarily assigned several chemicals that were detected and quantified in the extract could not be identified due to limited data (**B**, **C**, **F**, **M**, **N**, **Y** and **Z**). Some of them could be characterized as phenolic acid derivative based on the UV-Vis maxima observed in their spectra. During the analysis no flavonoids or their derivatives were detected in the analyzed composition (Figure 2, Table 2). All compounds most probably underwent decomposition under used chemical conditions.

The quantitative analysis of the altered extract (E2) was also performed. All detected compounds were quantified as chlorogenic acid equivalents. Undefined phenolic acid derivative (**B**) and chlorogenic acid (**I**) were dominating constituents (ca. 3.8 and 9.6 μg/mg). Significant content (ca. 6.2 μg/mg) of one of caffeoylquinic acid ethyl esters (**W**) was also established. The content of other detected chemicals varied from 0.06 to 1.30 μg/mg.

### 3.3. The Pharmacological Activity of the Extracts

MS is a complex of metabolic disorders characterized by insulin resistance, dyslipidemia, obesity and hypertension. The main diagnostic criteria include glucose and insulin levels, TAG and Ch-HDL. The data obtained in this work fully confirm the MS development in experimental animals. The results of the study of extracts pharmacological activity are presented in the Table 4. Keeping animals for six weeks on a diet enriched with fructose and saturated animal fats led to significant hyperglycemia, which accompanied by hyperinsulinemia, and indicated the IR state development. It is known that the fructose entry into the cell and its metabolic transformations are not controlled by insulin [34]. Under the excess intake of fructose by the organism, its metabolism in hepatocytes lead to the acetyl-CoA overproduction, which, in turn, will be involved in the lipogenesis process, and will be also utilized with the cholesterol synthesis. Subsequently, TAG and cholesterol are secreted by liver cells into the blood by VLDL formation. The data obtained in our study as for TAG and Ch-LDL elevated levels do not contradict literature evidence (Table 4).

Thus, keeping animals on HFD enriched with saturated triacylglycerols led to an increase in TAG level by 2.9 times (2.26 to 0.78 mmol/L), a decrease in Ch-HDL by 1.5 times (0.69 to 1.31 mmol/L) and an increase in Ch-LDL by 1.3 times (3.56 to 2.73 mmol/L) in rats with MS (Table 4). At the same time, increased fasting glucose (14.2 mmol/L) and insulin (3005 pg/mL) concentrations were observed in experimental animals from HFD group. This evidence of metabolic disorders indicates the development of experimental MS in animals, which also is verified by the elevated HOMA index [52]. Extract E1 administration in dose 150 mg/kg body weight caused a significant decrease in serum glucose (down to 11.4 mmol/L), while at doses of 250 mg/kg the effect was even more significant (down to 9.1 pg/mL). Additionally, the decrease in TAG concentration (down to 1.85 mmol/L) was noticed only in the case of the dose of 250 mg/kg (Table 4). The effects obtained with the introduction of E1 in dose of 350 mg/kg did not differ significantly from the effects observed with the introduction of 250 mg/kg. Administration of arginine-modified extract (E2) to animals with experimental MS at a dose of 250 mg/kg was accompanied by a decrease in glucose, insulin and TAG (down to 8.5 mmol/L, 2207 pg/mL and 1.56 mmol/L, respectively), as well as cholesterol metabolism alteration, thus, there was a decrease in Ch-LDL (down to 0.91 mmol/mL) and an increase in Ch-HDL (up to 3.31 mmol/L). Interestingly, the administration of E1_250 did not significantly improve Ch-LDL and Ch-HDL levels (Table 4). The values obtained for lower doses of E2 did not differ significantly from the effects of E2 extract at a dose of 350 mg/kg. The administration of arginine or quercetin at the dose of 250 mg/kg and 50 mg/kg, respectively to animals slightly influenced the levels of glucose, insulin and TAG, however, the observed effects were weaker than in the case of E1 and E2 (Table 4). 

## 4. Discussion

As a result of HPLC analysis of native aqueous-ethanolic extract from leaves of *V. corymbosum* 20 substances of phenolic nature were identified: 8 hydroxycinnamic acid derivatives and 12 flavonoids. Chlorogenic and neochlorogenic acids were shown to be dominant among hydroxycinnamic acids group while quercetin 3-*O*-galactoside (hyperoside), quercetin 3-*O*-rutinoside (rutin) and quercetin 3-*O*-glucoside (isoquercitrin) dominated among the flavonoids.

Due to its wide dietary use, majority of phytochemical studies were devoted to the *V. corymbosum* fruits composition. In highbush blueberry fruits, 44 flavonoids were previously identified, which were represented by quercetin and myricitin glycosides and chlorogenic acid [29,53]. The sugar part in the identified flavonoid glycosides from fruits was represented by glucose, galactose and arabinose [54], which corresponds to the results of our studies conducted for its leaves.

The previous phytochemical research devoted to the aerial parts of *V. corymbosum* L. allowed identification of isoquercitrin and rutin, caffeic and chlorogenic acids in methanolic extract from the leaves of plants cultivated in Romania [26]. Later studies on six samples of *V. corymbosum* L. leaves, cultivated in Romania confirmed the presence of rutin as a dominating flavonoid and feruloylquinic acid as dominating derivative of hydroxycinnamic acids group [16]. However, in our research hyperoside and chlorogenic acid were shown to be the major representatives of these groups what is in concordance with previous studies on the chlorogenic acid extraction *V. corymbosum* leaves using countercurrent chromatography [27].

Our research confirmed presence of previously identified phenolics in the plant materials obtained through cultivation of *V. corymbosum* in Romania such as chlorogenic and dicaffeoylquinic acid, rutin, isoquercitrin and avicularin [16]. What is more several compounds were identified, which were not previously found in *V. corymbosum*. They belong to hydroxycinnamic acid derivatives- 3-*O*-caffeoylquinic acid (neochlorogenic acid), 4-*O*-caffeoylquinic acid (cryptochlorogenic acid), *O*-mallonyl-*O*-caffeoylquinic acid, *O*-caffeoylshikimic acid, *O*-mallonyl-*O*-caffeoylquinic acid and *p*-coumaroyl-caffeoylquinic acid as well as flavonoids such as myricetin 3-*O*-galactoside, myricetin 3-*O*-glucoside, quercetin rhamnohexoside, kaempferol *O*-rhamnohexoside, quercetin *O*-malonylhexoside, mirycetin *p*-coumaroylhexoside and quercetin *p*-coumaroylhexoside were for the first time reported in *V. corymbosum* leaves.

The strategy of biologically active substances (BAS) modification by their conjugation with amino acids is an approach which has been already used for isolated molecules as well as their mixtures such as fractions or plant extracts. Modification of *β*-escin (mixture of horse chestnut triterpene saponins) with amino acid L-lysine led to a preparation known as L-lysine escinate, which is one of the main medicines for the treatment and prevention of strokes and peripheral circulatory disorders in Eastern Europe [9]. Modification of bearberry extract with phenylalanine resulted in more pronounced anti-inflammatory and diuretic activity [33]. Modification of motherwort tincture with different amino acids allowed to create new substances with stress-protective and anxiolytic properties [32]. Modification of the bilberry leaves extract with amino acid L-arginine allowed to create a substance with significant hypoglycemic and hypolipidemic activity [10]. Based on this experience, we obtained a complex of BAS with L-arginine basing on the highbush blueberry leaves extract which could, from the one side, allow better utilisation of this plant material from the other side lead to improvement of its biological properties which could be utilized in novel functional food development. The qualitative and quantitative analysis of the modified extract E2 showed significant changes in the chemical composition comparing to the unmodified extract E1. The content of chlorogenic acid decreased from 136.47 to 9.57 μg/mg, neochlorogenic—from 29.45 to 1.06 μg/mg, cryptochlorogenic—from 6.06 to 1.30 μg/mg, which is not proportional to the amount of arginine added. In comparison with E1 extract in E2 extract several new compounds were detected being classified as hydrolysis products, ethyl esters of hydroxycinnamic acids (substances **Q**, **R**, **S**, **W** and **X**), conjugates of phenolic substances with arginine (substances **H**, **K** and **L**) and a number of new undefined compounds (compound **N**, **Y** and **Z**). All this indicates changes in the composition of the extract E1 and the formation of conjugates with amino acids and appearance of new and modified principles, which could probably have a significant impact on the extract’s biological properties.

Medicinal correction of MS is primarily aimed at restoring cell sensitivity to insulin and improving of lipid metabolism. Excellent results in the metabolic disorders correction, which are characteristic for MS, have been reported using other polyphenol extracts [55]. It is well established that plant polyphenols show a wide range of effects, in particular, they reveal hypoglycemic, lipotropic, antioxidant, anti-inflammatory activity. The high content of chlorogenic acid in the extract prepared from *V. corymbosum* leaves, the presence of quercetin and kaempferol creates the necessary conditions for MS correction. The addition of arginine to E2 extract caused changes in its chemical composition resulting in the alteration of its anti-MS effects. Thus, arginine introduction in the incubation medium of L6 myotubes was shown to stimulate glucose uptake, glycogen formation, oxidation of palmitate by activating the Akt signaling pathway [56]. Similar effects were obtained in vivo, however, to obtain reliable results, arginine was administered in fairly high doses for 40 days or more [57]. That is why, in our opinion, the arginine administration in dose of 250 mg/kg for 14 days should not lead to significant changes. However, administration of extract modified by the arginine addition (E2_250) to animals with experimental MS allowed to maintain the positive effect of this compound without a significant increase in dose. E1 administration, already in dose 250 mg/kg body weight (HFD_E1_250) led to a significant decrease in the level of glucose, insulin and TAG in blood serum. At the same time, Ch-LDL and Ch-HDL content did not significantly change compered to HFD. The L-arginine modification of *V. corymbosum* leaf extract resulted in modification of its pharmacological properties. E2 had more pronounced normalizing effect as compared with the control group, seen as decrease of glucose and Ch-LDL levels, and improving of Ch-HDL. The results can be mediated by the complex effect of contained polyphenols, and the action of L-arginine, which is known to lower LDL levels, prevent LDL oxidation, and thus, expresses beneficial potential towards prevention of atherosclerosis [47].

Lifestyle adjustments, proper diet and the use of dietary supplements could significantly contribute to the reduction of the risk of metabolic syndrome development. Thus, it is recommended to include in diet a wide variety of food products, spices, omega fatty acids and substances rich in phenolics and their derivatives [2]. Several dietary factors are proven to prevent metabolic syndrome. Notable among these are olive oil, capsaicin, luteolin, curcumin, cinnamon, and rosemary, etc. A systemic review on the effects of dietary polyphenols on metabolic syndrome conducted recently showed that, at relatively high doses, many polyphenols favourably influence different health parameters associated with metabolic syndrome. Soy isoflavone, citrus products, hesperidin, and quercetin improved lipid metabolism, while cocoa supplementation improved high blood pressure and blood glucose levels. Consumption of green tea significantly reduced BMI and waist circumference and improved lipid metabolism [8]. It has been experimentally shown that blueberry and cranberry fruits in 18-year-old patients with type 2 diabetes significantly improve blood parameters [23]. *Vaccinium myrtillus* leaves extracts in reduced glucose levels, increased insulin sensitivity and normalized blood lipid profile in rats [10,11]. The obtained extracts E1 and E2 from the highbush blueberry leaves showed a lowering effect on glucose levels and normalized the lipid profile of the rats’ blood at the level which can be compared to the previously examined *Vaccinium myrtillus* extracts [10,11]. The extract E2 modified with arginine had a stronger effect and proved to be a promising agent for complex use in the prevention of insulin resistance, atherosclerosis and metabolic syndrome.

## 5. Conclusions

The conducted studies revealed potential of the phenolics contained in leaves of *V. corymbosum* in prevention of health conditions associated with metabolic syndrome which can be additionally augmented by their conjugation with L-arginine. These observations make a *V. corymbosum* leaves, being a by-product of a berry production, a good candidate for development of novel dietary supplements targeted on prevention of ailments associated with metabolic syndrome prevention.

## Figures and Tables

**Table 1:** Chemical composition of raw extract (E1) and quantification of major compounds using HPLC-DAD-MS.

No	Compound Name	Retention Time [min]	UV-Vis Maxima [nm]	MS^−^ Ions	MS^2−^ Ions	MS^3−^ Ions	MS+ Ions	MS^2+^ Ions	MS^3+^ Ions	Content (μg/mg)	Quantification Standard	Ions Used for Quantifiaction
1	3-*O*-caffeoylquinic acid (neochlorogenic acid) ^s^	16.2	242, 302, 324	353	191 b, 179, 135	-	355	163 b, 135	-	29.45 ± 0.57	chlorogenic acid	353, 707
2	5-*O*-caffeoylquinic acid (chlorogenic acid) ^s^	22.8	242, 300, 325	353	191 b, 179, 135	-	355	163	-	136.47 ± 4.26	chlorogenic acid	353, 707
3	4-*O*-caffeoylquinic acid (cryptochlorogenic acid)	24.7	300,324	353	191, 179, 173 b	-	355	163	-	6.09 ± 0.17	chlorogenic acid	353, 707
4	*O*-mallonyl-*O*-caffeoylquinic acid isomer	28.9	300, 324	439	233, 395 b, 353	-	441	423 b, 404, 163	-	17.85 ± 0.56	chlorogenic acid	439, 879
5	*O*-caffeoylshikimic acid isomer	30.5	300, 326	335	135, 179 b	-	337	163	-	2.53 ± 0.07	chlorogenic acid	335, 667
6	*O*-mallonyl-*O*-caffeoylquinic acid isomer	32.7	305, 326	439	233, 353, 395 b	-	441	163, 193, 244, 396, 423,645	-	13.07 ± 0.77	chlorogenic acid	439, 878
7	myricetin3-*O*-galactoside ^s^	36.3	265, 353	479	179, 316 b, 461	-	481	319	165	0.91 ± 0.03	hyperoside	479, 959
8	myricetin 3-*O*-glucoside ^s^	36.9	265, 353	479	179, 205, 297, 271, 316 b, 383, 461	-	481	319	-	1.03 ± 0.02	hyperoside	479, 959
9	quercetin rhamnohexoside	40.5	264, 353	609	301 b	-	611	303 b, 345	-	0.41 ± 0.01	hyperoside	609, 1219
10	quercetin 3-*O*-galactoside (hyperoside) ^s^	41.1	254, 262 sh, 353	463	301 b, 343, 179, 151, 229, 283, 255	-	465	**303 b**, 345	165 b, 195, 284	4.29 ± 0.10	hyperoside	463, 927
11	quercetin 3-*O*-rutinoside (rutin) ^s^	41.4	slope	609	301 b, 179, 271, 343, 395, 457	-	611	**465**, 303 b	447, 303 b	1.97 ± 0.05	hyperoside	609, 1219
12	quercetin 3-*O*-glucoside (isoquercitrin) ^s^	42.1	253, 264 sh, 353	463	301 b, 151, 179, 255, 273, 298, 344	-	465	303	-	2.33 ± 0.04	hyperoside	463, 927
13	quercetin 3-*O*-arabinoside (avicularin) ^s^	44.5	265, 354	433	301	-	435	303	137, 229 b, 257, 285	1.17 ± 0.04	hyperoside	433, 867
14	kaempferol *O*-rhamnohexoside	45.8	265, 342	593	199, 257, 285 b, 327, 447, 486, 565, 286 b	-	595	287 b, **449**	287	0.72 ± 0.01	hyperoside	593, 1187
15	quercetin *O*-malonylhexoside	46.1	264, 353	549	**505 b**, 301	301	551	303	-	0.26 ± 0.01	hyperoside	549, 1099
16	kaempferol 3-*O*-glucoside (astragalin) ^s^	46.5	265, 343	447	284	-	449	287 b, **303**	137, 191, 229 b	0.41 ± 0.01	hyperoside	447, 895
17	4,5-O-dicaffeoylquinic acid ^s^	51.0	328, 389	515	173, 179, 203, 255, 299, **353 b**, 404	135, 173 b	517	163, 296, 499 b	-	2.80 ± 0.30	chlorogenic acid	515, 1031
18	mirycetin *p*-coumaroylhexoside	52.6	259, 274, 281, 319, 321, 352	625	317, **479 b**	179, 316 b, 461	627	309, 319 b, 489, 609	-	0.36 ± 0.02	hyperoside	625, 1251
19	caffeic acid derivative	53.6	296, 324	207		-	209		-	1.44 ± 0.04	chlorogenic acid	207, 415
20	undefined phenolic acid	54.8	281, 335	451	341	-	453	191, 301, **343 b**, 435	191	0.58 ± 0.02	chlorogenic acid	451, 905
21	*p*-coumaroyl-caffeoylquinic acid	56.5	287, 315	499	173, **337 b**	173	501	321, **483 b**	147, 303 b	1.17 ± 0.20	chlorogenic acid	499, 999
22	quercetin *p*-coumaroylhexoside	58.0	256, 281, 321, 354	609	463, 301 b	-	303	165, 221, 267 b, 393, 428, 459	-	0.65 ± 0.02	hyperoside	609, 301

b—base peak (the most abundant ion in the recorded spectrum); in bold—ions subjected to MS^3^ fragmentation; ^s^—comparison of retention time with the standard was conducted; sh—shoulder in UV-vis spectrum.

**Table 2:** Chemical composition of extract conjugated with arginie (E2) and quantification of major compounds using HPLC-DAD-MS.

No	Compound Name	Retention Time [min]	UV-Vis Maxima [nm]	MS− Ions	MS^2−^ Ions	MS+ Ions	MS^2+^ Ions	Content/μg/mg	Qunatification Standard	Ions Used for Qunatification
A	quinic acid ^s^	1.4	227, 275	191	-	193	-	0.23 ± 0.21	chlorogenic acid	191
B	phenolic acid derivative	11.7	278	299	111, 173 b, 255	301	109, 224 b, 255	3.79 ± 0.05	chlorogenic acid	299
C	phenolic acid derivative	14.0	279, 311	299	109, 149, 173 b, 262, 281	301	-	0.06 ± 0.04	chlorogenic acid	299
D	3-*O*-caffeoylquinic acid (neochlorogenic acid) ^s^	16.3	300, 324	353	135, 179, 191 b	355	145, 163 b, 337	1.06 ± 0.20	chlorogenic acid	353, 707
E	3-O-*p*-coumaroylquinic acid	20.2	314	337	163 b, 173, 290	339	147 b	0.14 ± 0.02	chlorogenic acid	337
F	phenolic acid derivative	21.1	288, 326	533	179, 191, 353, 489 b	535	163, 355, 517 b	0.17 ± 0.08	chlorogenic acid	533
G	caffeic acid s	22.3	243, 300 sh, 324	179	-	709	355 b, 447, 499, 517, 691	0.27 ± 0.06	chlorogenic acid	179
H	caffeoylquinic acid-arginine conjugate I	22.5	280, 325	527	365, 353 b, 191	529	-	0.58 ± 0.14	chlorogenic acid	527
I	5-*O*-caffeoylquinic acid (chlorogenic acid) ^s^	22.9	245, 300 sh, 325	353	215, 191 b, 173	355	145, 163 b	9.57 ± 1.89	chlorogenic acid	353, 707
J	4-*O*-caffeoylquinic acid (cryptochlorogenic acid) ^s^	24.7	243, 300 sh, 326	353	173 b, 191	355	117, 163 b	1.30 ± 0.26	chlorogenic acid	353, 707
K	caffeoylquinic acid-arginine conjugate II	26.0	280, 324	527	179, 191 b, 353, 489	529	-	0.43 ± 0.02	chlorogenic acid	533
L	caffeoylquinic acid-arginine conjugate III	26.7	283, 325	527	353 b, 191	529	-	0.20 ± 0.05	chlorogenic acid	527
M	caffeoylquinic acid isomer	27.3	242, 300 sh, 324	353	215, 191 b, 173	353	-	0.15 ± 0.07	chlorogenic acid	353, 707
N	undefined compound	28.9	312	439	233, 395 b	441	-	0.12 ± 0.01	chlorogenic acid	439
O	caffeoylshikimic acid isomer	30.5	326	335	135, 179 b	337	114, 209, 322 b	0.18 ± 0.03	chlorogenic acid	335
P	caffeoylshikimic acid isomer	32.5	240, 324	335	135, 179 b	337	-	0.16 ± 0.02	chlorogenic acid	335
Q	dicaffeoylquinic acid ethyl ester isomer	32.7	280, 325	543	353 b, 335, 191	545	-	0.08 ± 0.04	chlorogenic acid	543
R	dicaffeoylquinic acid ethyl ester isomer	33.2	280, 324	543	353 b, 289, 191	545	-	0.26 ± 0.13	chlorogenic acid	543
S	caffeoylquinic acid ethyl ester isomer	34.1	243, 300 sh, 326	381	135, 161 b, 207, 335	383	-	0.52 ± 0.11	chlorogenic acid	381, 763
T	caffeoylshikimic acid isomer	34.5	243, 300 sh, 323	335	135, 179 b	337		0.11 ± 0.01	chlorogenic acid	335
U	caffeoylquinic acid ethyl ester isomer	39.7	243, 300 sh, 326	381	161 b, 207, 335	383	163 b, 221	0.56 ± 0.14	chlorogenic acid	381, 763
W	caffeoylquinic acid ethyl ester isomer	42.3	243, 300 sh, 326	381	135, 179 b, 191	383	145, 163 b, 221, 365	6.23 ± 0.97	chlorogenic acid	381, 763
X	caffeoylquinic acid ethyl ester isomer	45.5	243, 300 sh, 326	381	135, 179 b, 191, 335	383	163b, 221	0.07 ± 0.01	chlorogenic acid	381, 763
Y	undefined compound	51.4	370	489	243, 269, 287 b	-	-	0.15 ± 0.04	chlorogenic acid	489
Z	undefined compound	53.5	293, 333	207	-	227	-	0.24 ± 0.06	chlorogenic acid	207

b—base peak (the most abundant ion in the recorded spectrum); ^s^—comparison of retention time with the standard was conducted; sh—shoulder in UV-Vis spectrum.

**Table 3:** The quantitative content of phenolic compounds in highbush blueberry leaves dry extracts (M ± m, *n* = 6).

Phytochemical Group	Method Used	Content, %	
		Extract 1 (E1)	Extract 2 (E2)
hydroxycinnamic acids derivatives	spectrophotometric as chlorogenic acid equivalents (λ = 327 nm)	2.92 ± 0.12 #	1.82 ± 0.02
flavonoids	spectrophotometric as rutin equivalents (λ = 417 nm)	3.03 ± 0.11 #	1.96 ± 0.05
total phenolics	spectrophotometric as gallic acid equivalents (λ = 270 nm)	18.42 ± 0.97 #	12.09 ± 0.07

#—statistically significant difference between extracts, *p* < 0.05.

**Table 4:** The hypoglycemic activity of the investigated extracts under of high-fructose diet (M ± m, *n* = 6). HFD—high fructose diet group; HFD_E1—high fructose diet group supplemented with extract 1 (E1); HFD_E2—high fructose diet group supplemented with extract 2 (E2); HFD_E2—high fructose diet group supplemented with extract 2 (E2); HFD_Arg —high fructose diet group supplemented with arginine; HFD_Q—high fructose diet group supplemented with quercetin; *—indicates significant difference relative to normal (*p* < 0.05); #—indicates significant difference relative to HFD (*p* < 0.05).

Treatment	Glucose, mmol/L	Insulin, pg/mL	Triacylglycerols (TAG), mmol/L	Ch-HDL, mmol/L	Ch-LDL, mmol/L
normal	4.4 ± 0.09	1199 ± 25	0.78 ± 0.03	1.31 ± 0.03	2.73 ± 0.06
HFD	14.2 ± 0.19 *	3005 ± 48 *	2.26 ± 0.06 *	0.69 ± 0.03 *	3.56 ± 0.06 *
HFD_E1_150 mg/kg	11.4 ± 0.17 *#	2986 ± 37 *	2.23 ± 0.12 *	0.77 ± 0.11 *	3.05 ± 0.09 *
HFD_E1_250 mg/kg	9.1 ± 0.10 *#	2347 ± 21 *#	1.85 ± 0.09 *#	0.79 ± 0.02 *	3.44 ± 0.14 *
HFD_E1_350 mg/kg	9.0 ± 0.21 *#	2325 ± 35 *#	1.93 ± 0.15 *#	0.75 ± 0.07 *	3.35 ± 0.07 *
HFD_E2_150 mg/kg	11.6 ± 0.19 *#	2793 ± 41 *#	1.96 ± 0.12 *	0.79 ± 0.08 *#	3.41 ± 0.12 *
HFD_E2_250 mg/kg	8.5 ± 0.18 *#	2207 ± 19 *#	1.56 ± 0.09 *#	0.93 ± 0.08 *#	3.31 ± 0.10 *#
HFD_E2_350 mg/kg	8.7 ± 0.15 *#	2211 ± 23 *#	1.63 ± 0.13 *#	0.91 ± 0.08 *#	3.29 ± 0.09 *
HFD_Arg_250 mg/kg	10.7 ± 0.25 *	2604 ± 32 *	1.98 ± 0.06 *	0.73 ± 0.05 *	3.58 ± 0.08 *
HFD_Q_50 mg/kg	11.2 ± 0.10 *#	2734 ± 19 *#	2.24 ± 0.18 *	0.80 ± 0.08 *	3.39 ± 0.11 *

## Data Availability

Not applicable.
